# Improving EEG-Based Driver Fatigue Classification Using Sparse-Deep Belief Networks

**DOI:** 10.3389/fnins.2017.00103

**Published:** 2017-03-07

**Authors:** Rifai Chai, Sai Ho Ling, Phyo Phyo San, Ganesh R. Naik, Tuan N. Nguyen, Yvonne Tran, Ashley Craig, Hung T. Nguyen

**Affiliations:** ^1^Faculty of Engineering and Information Technology, Centre for Health Technologies, University of TechnologySydney, NSW, Australia; ^2^Data Analytic Department, Institute for Infocomm ResearchA*STAR, Singapore, Singapore; ^3^Kolling Institute of Medical Research, Sydney Medical School, The University of SydneySydney, NSW, Australia

**Keywords:** electroencephalography, driver fatigue, autoregressive model, deep belief networks, sparse-deep belief networks

## Abstract

This paper presents an improvement of classification performance for electroencephalography (EEG)-based driver fatigue classification between fatigue and alert states with the data collected from 43 participants. The system employs autoregressive (AR) modeling as the features extraction algorithm, and sparse-deep belief networks (sparse-DBN) as the classification algorithm. Compared to other classifiers, sparse-DBN is a semi supervised learning method which combines unsupervised learning for modeling features in the pre-training layer and supervised learning for classification in the following layer. The sparsity in sparse-DBN is achieved with a regularization term that penalizes a deviation of the expected activation of hidden units from a fixed low-level prevents the network from overfitting and is able to learn low-level structures as well as high-level structures. For comparison, the artificial neural networks (ANN), Bayesian neural networks (BNN), and original deep belief networks (DBN) classifiers are used. The classification results show that using AR feature extractor and DBN classifiers, the classification performance achieves an improved classification performance with a of sensitivity of 90.8%, a specificity of 90.4%, an accuracy of 90.6%, and an area under the receiver operating curve (AUROC) of 0.94 compared to ANN (sensitivity at 80.8%, specificity at 77.8%, accuracy at 79.3% with AUC-ROC of 0.83) and BNN classifiers (sensitivity at 84.3%, specificity at 83%, accuracy at 83.6% with AUROC of 0.87). Using the sparse-DBN classifier, the classification performance improved further with sensitivity of 93.9%, a specificity of 92.3%, and an accuracy of 93.1% with AUROC of 0.96. Overall, the sparse-DBN classifier improved accuracy by 13.8, 9.5, and 2.5% over ANN, BNN, and DBN classifiers, respectively.

## Introduction

Fatigue during driving is a major cause of road accidents in transportation, and therefore poses a significant risk of injury and fatality, not only to the drivers themselves but also to other road users such as passengers, motorbike users, other drivers, and pedestrians (Matthews et al., 2012). Driver fatigue reduces the ability to perform essential driving skills such as vehicle steering control, tracking vehicle speed, visual awareness, and sufficient selective attention during a monotonous driving condition for a long period of time (Lal and Craig, 2001; Wijesuriya et al., 2007; Craig et al., 2012; Jurecki and Stańczyk, 2014). As a result an automated countermeasure for a driver fatigue system with reliable and improved fatigue classification/detection accuracy is needed to overcome the risk of driver fatigue in transportation (Lal et al., 2003; Vanlaar et al., 2008; Touryan et al., 2013, 2014; Chai et al., 2016).

In the digital age, machine learning can be used to provide automated prediction of driver fatigue. Two approaches can be used in machine learning, which are the regression and classification methods. The goal of regression algorithms is the prediction of continuous values to estimate driving performance (Lin et al., 2005; Touryan et al., 2013, 2014). The outcome of classification algorithms is to predict the target class, such as the classification between fatigue and non-fatigue/alert states (Lin et al., 2010; Zhang et al., 2014; Chai et al., 2016; Xiong et al., 2016). The aim of this study is to improve the accuracy of the prediction of fatigue and non-fatigue states. As a result, this study focuses on using an advanced classification method for enhancing the accuracy of a fatigue classification system previously studied (Chai et al., 2016).

As described in a previous paper (Chai et al., 2016), possible driver fatigue assessment includes psychological and physiological measurements (Lal and Craig, 2001; Borghini et al., 2014). For instance, psychological measurement of driver fatigue involves the need for frequent self-report of fatigue status via brief psychometric questionnaires (Lai et al., 2011). Such an approach would be difficult to implement and may well be biased given its subjective nature (Craig et al., 2006). Physiological measurement of the driver fatigue includes video measurement of the face (Lee and Chung, 2012), brain signal measurement using electroencephalography (EEG; Lal et al., 2003; Lin et al., 2005; Craig et al., 2012; Chai et al., 2016), eye movement tracking system using camera and electrooculography (EOG; Hsieh and Tai, 2013), and heart rate measurement using electrocardiography (ECG; Tran et al., 2009; Jung et al., 2014).

Physiological assessment of facial or eye changes using video recording of the driver's face may lead to privacy issues. Physiological measurement strategies like monitoring eye blink rates using EOG and heart rate variability (HRV) using ECG have been shown to reliably detect fatigue (Tran et al., 2009; Hsieh and Tai, 2013). EEG has also been shown to be a reliable method of detecting fatigue, as it directly measures neurophysiological signals that are correlated with mental fatigue (Wijesuriya et al., 2007; Craig et al., 2012; Zhang et al., 2014; Chuang et al., 2015; He et al., 2015; Xiong et al., 2016). Recently, we have shown a classification of EEG-based driver fatigue with the inclusion of new ICA based pre-processing with a promising classification result (Chai et al., 2016), however, it was concluded the classification accuracy needs to be improved. As a result, this paper will extend the work on a potential EEG-based countermeasure driver fatigue system with an improved classification of fatigue vs. alert states.

An EEG-based classification countermeasure system requires several components including EEG signal measurement, signal pre-processing, feature extraction, and classification modules. For feature extraction in EEG analysis, frequency domain data has been widely explored (Lal and Craig, 2001; Craig et al., 2012). Power spectral density (PSD) methods are popular for converting the time domain of EEG signal into the frequency domain (Demandt et al., 2012; Lin et al., 2014). Alternatively, an autoregressive (AR) modeling parametric approach can also be used for feature extraction in an EEG classification system (McFarland and Wolpaw, 2008; Chai et al., 2016; Wang et al., 2016). The advantage of AR modeling is its inherent capacity to model the peak spectra that are characteristic of the EEG signals and it is an all-pole model making it efficient for resolving sharp changes in the spectra. In our previous finding, an AR modeling feature extractor provided superior classification results compared to PSD for EEG-based driver fatigue classification (Chai et al., 2016). Therefore, in this paper, we present the results of applying AR for modeling feature extraction in order to improve the accuracy the classification algorithm. The PSD method is also included for comparison. For the classification, non-linear methods, such as artificial neural networks (ANN), have been used widely in a variety of applications involving EEG (Nguyen, 2008; Casson, 2014). Bayesian neural networks (BNN) have also been used in EEG-based driver fatigue classification (Chai et al., 2016). The Bayesian regularization framework is able to enhance the generalization of neural networks training regardless of finite and/or noisy data.

Recent attention has been focused on improvement of an ANN approach called deep belief networks (DBN; Hinton and Salakhutdinov, 2006; Hinton et al., 2006; Bengio, 2009; LeCun et al., 2015), which involves a fast, unsupervised learning algorithm for the deep generative model, and supervised learning for a discriminative model. The key advantage of this algorithm is the layer-by-layer training for learning a deep hierarchical probabilistic model efficiently as well as a discriminative fine tuning algorithm to optimize performance on the classification problems (Bengio, 2009; LeCun et al., 2015). A DBN classifier is a promising strategy for improving classification of problems including hand-writing character classification (Hinton et al., 2006), speech recognition (Mohamed et al., 2010; Hinton et al., 2012), visual object recognition (Krizhevsky et al., 2012), and other biomedical applications (O'Connor et al., 2013; Stromatias et al., 2015). The training of the DBN is based on the restricted Boltzmann machine (RBM) with layers-wise training of the network per layer at a time from the bottom up (Hinton et al., 2006). Furthermore, the original RBM approach tended to learn a distributed non-sparse representation. A modified version of the RBM using sparse-RBM to form a sparse-deep belief network (sparse-DBN) has shown promising results for modeling low-order features as well as higher-order features for the application of image classification with improved accuracy (Lee et al., 2008; Ji et al., 2014). As a result of this promising advance in classification of complex features, this paper further investigates the classification of EEG signals associated with driver fatigue using the sparse-DBN. For comparison purposes, the results from several different classifiers are included to determine which algorithms are superior with the highest classification performance.

The main contribution of this paper is the combination of the AR modeling feature extractor and sparse-DBN classifier which have not been explored previously for EEG-based driver fatigue classification, with the objective of enhancing the classification performance over past attempts (Chai et al., 2016). The motivation to utilize the sparse-DBN classifier was to investigate its potential superiority for classifying fatigue, in comparison to other classifiers. Sparse-DBN is a semi supervised learning method that combines unsupervised learning for modeling the feature in the pre-training layer and supervised learning for discriminating the feature in the following layer. Incorporating the sparsity in sparse-DBN, achieved with a regularization term that penalizes a deviation of the expected activation of hidden units from a fixed low-level, prevents the network from overfitting, and is able to learn low-level structures as well as high-level structures (Ji et al., 2014).

## Background and methodology

### General structure

**Figure 1:**
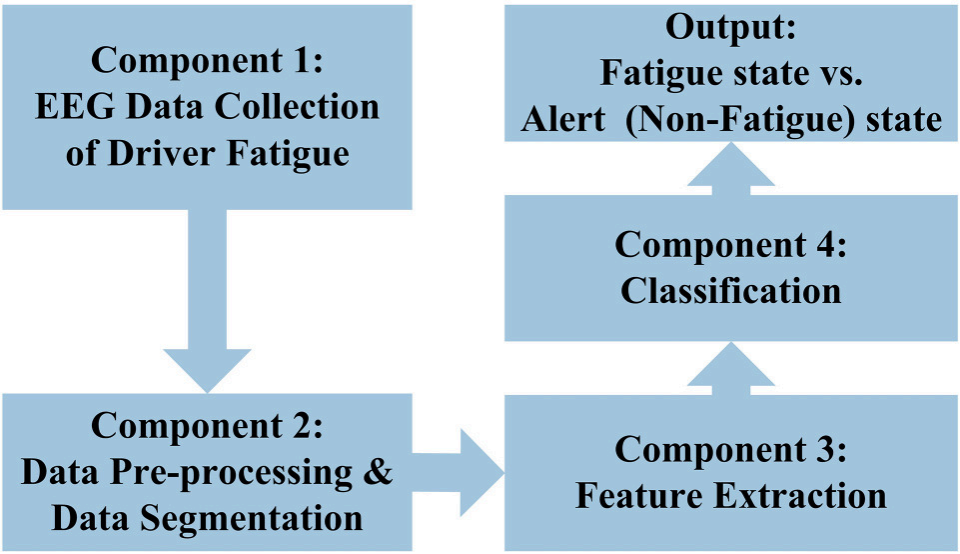
**General structure EEG-based driver fatigue classification in this study**.

The general structure for the EEG-based driver fatigue classification used in this paper is shown in Figure 1 which is divided into four components: (i) the first component involves EEG data collection in a simulated driver fatigue environment; (ii) the second component involves data pre-processing for removing EEG artifact and the moving window segmentation; (iii) the third component involves the features extraction module that converts the signals into useful features; (iv) the fourth component involves the classification module to process the feature and which translates into output via training and classification procedures. The output of the classification comprises two states: fatigue state and alert (non-fatigue) state.

### EEG data collection

The EEG data collection has been described in a previous paper (Chai et al., 2016). The study was approved by the Human Research Ethics Committee of the University of Technology Sydney (UTS) obtained from previous experiments of driver fatigue study (Craig et al., 2006, 2012; Wijesuriya et al., 2007). The dataset contains electrophysiological data from 43 healthy participants aged between 18 and 55 years who had a current driver's license. The study involved continuous measurement taken during a monotonous simulated driving task followed by post-EEG measures and post-subjective self-report of fatigue. For the simulated driving task, the divided attention steering simulator (DASS) from Stowood scientific instruments was used (Craig et al., 2012). Participants were asked to keep driving at the center of the road in the simulation task. The participants were also required to respond to a target number that appeared in any of the four corners of the computer screen in front of the participants when they were driving in the experiment, so as to record reaction time.

The simulation driving task was terminated if the participant drove off the simulated road for >15 s, or if they showed consistent facial signs of fatigue such as head nodding and extended eyes closure, both determined by analysis of participants' faces that occurred throughout the experiment. Three methods were used to validate fatigue occurrence: (i) using video monitoring for consistent physiological signs of fatigue such as tired eyes, head nodding and extended eye closure, verified further by EOG analysis of blink rate and eye closure; (ii) using performance decrements such as deviation off the road, and (iii) using validated psychometrics such as the Chalder Fatigue Scale and the Stanford Sleepiness Scale. Two participants who did not meet the criterion of becoming fatigued were excluded from the dataset. The validation of fatigue vs. non-fatigue in these participants has been reported in prior studies (Craig et al., 2006, 2012). The EEG signals were recorded using a 32-channel EEG system, the Active-Two system (Biosemi) with electrode positions at: FP1, AF3, F7, F3, FC1, FC5, T7, C3, CP1, CP5, P7, P3, PZ, PO3, O1, OZ, O2, PO4, P4, P8, CP6, CP2, C4, T8, FC6, FC2, F4, F8, AF4, FP2, FZ, and CZ. The recorded EEG data was down sampled from 2,048 to 256 Hz.

### Data pre-processing and segmentation

For the alert status, the first 5 min of EEG data was selected when the driving simulation task began. For the fatigue status, the data was selected from the last 5 min of EEG data before the task was terminated, after consistent signs of fatigue were identified and verified. Then in each group of data (alert and fatigue), 20 s segments were taken with the segment that was chosen being the first 20 s where EEG signals were preserved. For the sample this was all within the first 1 min of the 5 min selected. Further, artifact removal using an ICA-based method was used to remove blinks, heart, and muscle artifact. As a result, 20 s of the alert state and 20 s of the fatigue state data were available from each participant.

**Figure 2:**
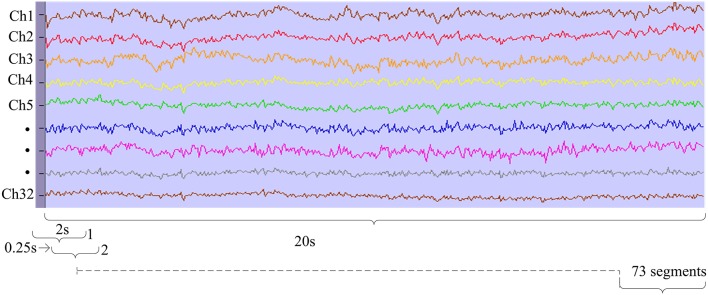
**Moving window segmentation for driver fatigue study**.

In the pre-processing module before feature extraction, the second-order blind identification (SOBI) and canonical correlation analysis (CCA) were utilized to remove artifacts of the eyes, muscle, and heart signals. The pre-processed data were segmented by applying a moving window of 2 s with overlapping 1.75 s to the 20 s EEG data which provided 73 overlapping segments for each state (fatigue and alert states) as shown in Figure 2. The pre-processing segments were used in the feature extraction module as described in next section.

### Feature extraction

For comparison purposes and validity of previous work, a feature extractor using the power spectral density (PSD), a widely used spectral analysis of feature extractor in fatigue studies, is provided in this paper.

An autoregressive (AR) model was also applied as a features extraction algorithm in this study. AR modeling has been used in EEG studies as an alternative to Fourier-based methods, and has been reported to have improved classification accuracy in previous studies compared to spectral analysis of the feature extractor (Brunner et al., 2011; Chai et al., 2016). The advantage of AR modeling is its inherent capacity to model the peak spectra that are characteristic of the EEG signals and it is an all-pole model making it efficient for resolving sharp changes in the spectra. The fast Fourier transform (FFT) is a widely used non-parametric approach that can provide accurate and efficient results, but it does not have acceptable spectral resolution for short data segments (Anderson et al., 2009). AR modeling requires the selection of the model order number. The best AR order number requires consideration of both the signal complexity and the sampling rate. If the AR model order is too low, the whole signal cannot be captured in the model. On the other hand, if the model order is too high, then more noise is captured. In a previous study, the AR order number of five provided the best classification accuracy (Chai et al., 2016). The calculation of the AR modeling was as follows:

(1) $$\hat{x}(t)=\underset{k\;=\;1}{\sum^{P}}a(k)\hat{x}(t-k)+e(t)$$

where $\hat{x}\left(t\right)$ denotes EEG data at time (*t*), *P* denotes the AR order number, *e*(*t*) denotes the white noise with zero means error and finite variance, and *a(k)* denotes the AR coefficients.

### Classification algorithm

The key feature of DBN is the greedy layer-by-layer training to learn a deep, hierarchical model (Hinton et al., 2006). The main structure of the DBN learning is the restricted Boltzmann machine (RBM). A RBM is a type of Markov random field (MRF) which is a graphical model that has a two-layer architecture in which the observed data variables as visible neurons are connected to hidden neurons. A RBM is as shown in which m visible neuron [*v* = (*v*_1_, *v*_2_, *v*_3_,…,*v*_*m*_)] and *n* hidden neurons [*h* = (*h*_1_, *h*_2_,…, *h*_*n*_)] are fully connected via symmetric undirected weights and there is no intra-layer connections within either the visible or the hidden layer.

The connections weights and the biases define a probability over the joint states of visible and hidden neurons through energy function *E*(*v,h*), defined as follows:

(2) $$E\left(v,h;\theta \right)=-\underset{i\;=\;1}{\sum^{m}}\underset{j\;=\;1}{\sum^{n}}w_{ij}v_{i}h_{j}-\underset{i\;=\;1}{\sum^{m}}a_{i}v_{i}-\underset{j\;=\;1}{\sum^{n}}b_{j}h_{i}$$

where *w*_*ij*_ denotes the weight between *v*_*i*_ and *h*_*j*_ for all *i* Î {1,…, *m*} and *j* Î {1,…, *n*}; *a*_*i*_ and *b*_*j*_ are the bias term associated with the *ith* and *jth* visible and hidden neurons; θ = {*W*,*b*,*a*} is the model parameter with symmetric weight parameters *W*_*nm*_.

For RBM training, the gradient of log probability of a visible vector (*v*) over the weight *w*_*ij*_ with the updated rule calculated by constructive divergence (CD) method is as follows:

(3) $$\Delta w_{ij}=\eta \left({\langle v_{i}h_{j}\rangle }_{data}-{\langle v_{i}h_{j}\rangle }_{recon}\right)$$

where η is a learning rate, 〈*v_i_h_j_*〉*_recon_* is the reconstruction of original visible units which is calculated by setting the visible unit to a random training vector. The updating of the hidden and visible states is considered as follows:

(4) $$p\left(h_{j}=1\;|\;v\right)=\sigma \left(b_{j}+\sum_{i}v_{i}w_{ij}\right)$$

(5) $$p\left(v_{i}=1\;|\;h\right)=\sigma \left(a_{i}+\sum_{i}h_{j}w_{ij}\right)$$

where σ is the logistic sigmoid function.

The original RBM tended to learn a distributed, non-sparse representation of the data, however sparse-RBM is able to play an important role in learning algorithms. In an information-theoretic sense, sparse representations are more efficient than the non-sparse ones, allowing for varying of the effective number of bits per example and able to learn useful low- and high-level feature representations for unlabeled data (i.e., unsupervised learning; Lee et al., 2008; Ji et al., 2014).

**Figure 3:**
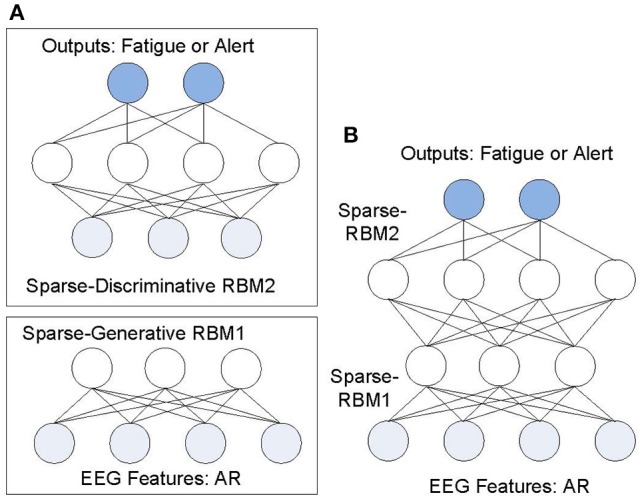
**Structure of sparse-DBN for driver fatigue classification: (A)** Greedy learning stack of sparse-RBM; **(B)** the corresponding sparse-DBN.

This paper uses the sparse-RBM to form the sparse-DBN for EEG-based driver fatigue classification. The sparsity in sparse-DBN is achieved with a regularization term that penalizes a deviation of the expected activation of hidden units from a fixed low-level, which prevents the network from overfitting, as well as allowing it to learn low-level structures as well as high-level structures (Ji et al., 2014). The sparse-RBM is obtained by adding a regularization term to the full data negative log likelihood with the following optimization:

(6) $$\begin{array}{l} \underset{\left\{w_{ij}a_{i}b_{j}\right\}}{\min}E\left(v,h,\theta \right)-\underset{l\;=\;1}{\sum^{m}}\;log\underset{h}{\;\sum }P\left(v^{\left(l\right)},h^{\left(l\right)}\right)\\ \;+\lambda \underset{j\;=\;1}{\sum^{n}}{\left|p-\frac{1}{m}\underset{l\;=\;1}{\sum^{m}}𝔼\left[h_{j}^{\left(l\right)}|v^{\left(l\right)}\right]\right|}^{2} \end{array}$$

where 𝔼[.] is the conditional expectation given the data, λ is a regularization constant, and *p* is a constant controlling the sparseness of the hidden neurons *h*_*j*_. The DBN is constructed by stacking a predefined number of RBMs to allow each RBM model in the sequence to receive a different representation of the EEG data. The modeling between visible input (*v*) and *N* hidden layer *h*_*k*_ is as follows:

(7) $$P\left(v,h^{1},\ldots ,h^{l}\right)=\left(\underset{k\;=\;0}{\prod^{l-2}}P\left(\left[h^{\left(k\right)}|h^{\left(k+1\right)}\right]\right)\right)P\left(h^{l-1},h^{l}\right)$$

where *v* = *h*^0^, *P*(*h*^*k*^|*h*^*k*+1^) is a conditional distribution for the visible units conditioned on the hidden units of the RBM at level *k* and *P*(*h*^*l*−1^,*h*^*l*^) is the visible-hidden joint distribution at the top-level RBM. Two training types of the RBM can be used: generative and discriminative. The generative training of RBM is used as pre-training with un-supervised learning rule. After greedy layer-wise unsupervised learning, the DBN can be used for discriminative ability using the supervised learning. This paper uses a sparse variant of DBN with 2 layers of semi supervised sparse-DBN as shown in Figure 3 with the first layer using the sparse-RBM for generative mode (un-supervised learning) and the second layer using the sparse-RBM in discriminative mode (supervised learning). After layer-by-layer training in DBN, an ANN with back-propagation method is used through the whole classifier to fine-tune the weights for optimal classification.

The performance indicators, including, sensitivity or true positive rate [*TPR* = *TP*/(*TP*+*FN*)], specificity or true negative rate [*TNR* = *TN*/(*TN*+*FP*)] and accuracy (TP+TN)/(TP+TN+FP+FN), were used for the performance measurement. *TP* (true positive) denotes the number of the fatigue data correctly classified as fatigue state. *FP* (false positive) is the number of alert datasets classified as a fatigue state. *TN* (true negative) is number of alert datasets correctly classified as an alert state. *FN* (false negative) is the fatigue datasets classified as an alert state.

For network learning generalization, we presented the results based on two cross-validation techniques: an early stopping technique and *k*-fold cross-validation. The early stopping technique used the “hold-out cross validation”—one of the widely used cross validations techniques. Basically, it divided the dataset into three subsets (training, validation, and testing sets). The model is trained using the training set while the validation set is periodically used to evaluate the model performance to avoid over-fitting/over-training. The accuracy of the testing set is used as the result of the model's performance. Another cross validation technique is known as *k*-fold cross-validation (*k* = 3). In *k*-fold cross-validation (*k* = 3), the dataset is divided into three equal (or near equal) sized folds. The training of the network uses 2-folds and the testing the network uses the remaining fold. The process of training and testing is repeated for three possible choices of the subset omitted from the training. The average performance on the three omitted subsets is then used as an estimate of the generalization performance.

Furthermore, a receiver operating characteristic (ROC) graph is used to evaluate further the performance of the proposed method with the compared method for this study. The areas under the curve of the ROC (AUROC) were also computed to evaluate quantitatively the classification performance.

## Results

From the 32-EEG channel dataset for the 43 participants (2 participants who did not meet the criterion of becoming fatigued were excluded from original 45 participants), 20 s of alert state and 20 s of fatigue state data were available from each participant. This was fed to the pre-processing module including artifact removal and a 2 s moving window segmentation with overlapping 1.75 s to the 20 s EEG data, providing 73 overlapping segments for each state. As a result, from the 43 participants, a total 6,278 units of datasets were formed for the alert and fatigue states (each state having 3,139 units).

The segmented datasets were fed to the feature extraction module. AR modeling with the order number of 5 was used for the feature extractor as it provided an optimum result from the previous study (Chai et al., 2016). The size of the AR features equaled the AR order number multiplied with 32 units of EEG channels, thus the AR order number of 5 resulted in 160 units of the AR features. For comparison and validity purposes, this paper includes the PSD, a popular feature extractor in the EEG classification for driver fatigue classification. The spectrum of EEG bands consisted of: delta (0.5–3 Hz), theta (3.5–7.5 Hz), alpha (8–13 Hz), and beta activity (13.5–30 Hz). The total power for each EEG activity band was used for the features that were calculated using the numerical integration trapezoidal method, providing 4 units of power values. This resulted in 128 units of total power of PSD for the 32 EEG channels used.

The variant of standard DBN algorithm, sparse-DBN with semi supervised learning used in this paper, comprised of one layer of sparse-RBM with the generative type learning and the second layer of sparse-RBM with discriminative type of learning. The training of the sparse-DBN is done layer-by-layer. The ANN with back-propagation method was used to fine-tune the weights for optimal classification.

For the discriminative learning of sparse-DBN, the total 6,278 datasets were divided into three subsets with similar amounts of number sets: training (2,093 sets) validation (2,093 sets), and testing sets (2,092 sets). The generative learning of sparse-DBN uses unlabeled data from the training sets. For the training of the sparse-DBN using the learning rate (η) of 0.01, the maximum epoch is set to 200, with a regularization constant (λ) of 1, and the constant controlling the sparseness (*p*) of 0.02. The selection of these training parameters was chosen by trial-and-error, with the chosen values achieving the best training result. Table 1 shows the selection of the regularization constant (λ), with the chosen value of 1 and the constant controlling the sparseness (*p*) with the chosen value of 0.02, providing lowest the mean square error (MSE) values of 0.00119 (training set) and 0.0521 (validation set) with the iteration number of 69. The average of the MSE values was 0.0046 ± 0.0018 (training set), and 0.0760 ± 0.0124.

**Table 1:** **Testing several values of regularization constant (λ) and the constant controlling the sparseness (***p***) in order to select values with the lowest MSE (trial-and-error method)**.

**Regularization constant*(λ)***	**Sparseness constant *(p)***	**MSE training**	**MSE validation**	**Iteration number**
0.5	0.1	0.00492	0.06625	90
1	0.1	0.00680	0.06710	82
2	0.1	0.00676	0.07961	64
0.5	0.01	0.00542	0.07365	66
1	0.01	0.00507	0.08360	71
2	0.01	0.00395	0.06831	85
0.5	0.02	0.00288	0.07664	73
**1**	**0.02**	**0.00119**	**0.05206**	**69**
2	0.02	0.00288	0.07181	66
0.5	0.03	0.00327	0.08289	88
1	0.03	0.00574	0.09207	73
2	0.03	0.00665	0.09825	89
Mean		0.004629	0.07615	76.42
*SD*		0.001803	0.01269	9.72

*Bold values signify the chosen parameters*.

**Figure 4:**
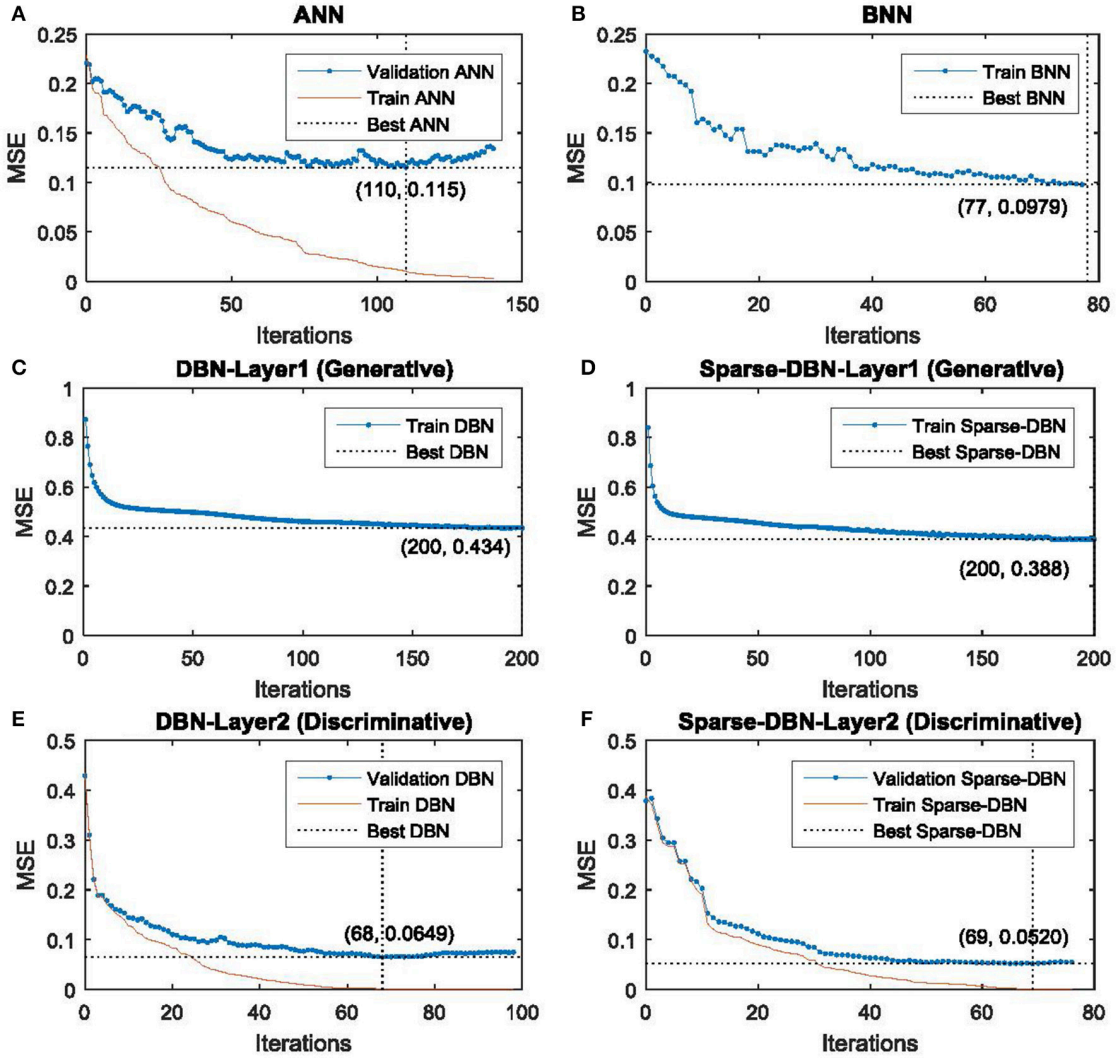
**Plot of the training and validation MSE for early stopping of classifiers: (A)** MSE training and validation of ANN. **(B)** MSE training of BNN. **(C)** MSE training of DBN in hidden layer 1 (Generative mode). **(D)** MSE training of sparse-DBN in hidden layer 1 (Generative mode). **(E)** MSE training and validation of DBN in hidden layer 2 (Discriminative mode). **(F)** MSE training and validation of DBN in hidden layer 2 (Discriminative mode).

In order to prevent over-fitting/over-training in the network, a validation-based early stopping method was used for the proposed classifier of sparse-DBN. The plot of the mean square error (MSE) training set and validation set are shown in Figure 4 for classification using AR and sparse-DBN. Table 2 shows the best performance of the training in term of the MSE values and iteration numbers. For comparison, the results for ANN, BNN, and DBN classifier are also included.

**Table 2:** **The best MSE and iteration numbers from the training of the classifiers (ANN, BNN, DBN, and Sparse-DBN)**.

**Classifiers**	**Best MSE**	**Best iteration number**
ANN	0.115	110
BNN	0.0979	77
DBN	0.0649	68
Sparse-DBN	0.0520	69

ANN, DBN and sparse-DBN classifiers utilized the early stopping framework (with the dataset divided into training validation and test sets) to prevent the overfitting problem, except for BNN (where the dataset was divided into training and testing). The BNN used a different framework for preventing the overfitting problem utilizing adaptive hyper-parameters in the cost function to prevent the neural network weight from being too large, which would have resulted in poor generalization. As a result, the validation set is not required for the BNN. A detailed analysis of BNN for EEG based driver fatigue classification has been addressed in our previous study (Chai et al., 2016). The core parameters for the training classifiers (ANN, BNN, DBN, and sparse-DBN) are the ANN-based classifier which includes the number of hidden nodes, an activation function, and learning rate. In the BNN classifier, an additional hyper-parameter is introduced to fine tune the optimal structure of the ANN. Further, in the sparse-DBN classifier, the regulation constant and constant controlling of sparseness were introduced for the training the DBN classifier. The DBN and sparse-DBN used two hidden layers: the first hidden layer as generative mode (un-supervised learning) and second hidden layer as discriminative mode (supervised learning).

The mean square error (MSE) of the training set decreased smoothly. Using ANN classifier, the training network stopped after 100 iterations as the MSE validation set reached a maximum fail of 10 times the increment value to ensure no over-training happened with the best validation MSE at 0.115. Using a BNN classifier, the training network stopped after 77 iterations as the conditions are met with the BNN parameters with the best validation MSE at 0.0979. Using a DBN classifier in the first hidden layer (generative mode), the training network stopped after 200 iterations with best MSE at 0.434. Using a DBN classifier in the second hidden layer (discriminative mode), the training network stopped after 68 iterations as the MSE validation set reached maximum fail of 10 times increment value to ensure no over-training happened with the best validation MSE at 0.0649. Using the proposed method of sparse-DBN classifier in the first hidden layer (generative mode), the training network stopped after 200 iterations with the best of MSE at 0.388. Using the proposed method of sparse-DBN classifier in the second hidden layer (discriminative mode), the training network stopped after 69 iterations as the MSE validation set reached maximum fail of 10 times increment value to ensure no over-training happened, with the best validation MSE at 0.0520.

**Figure 5:**
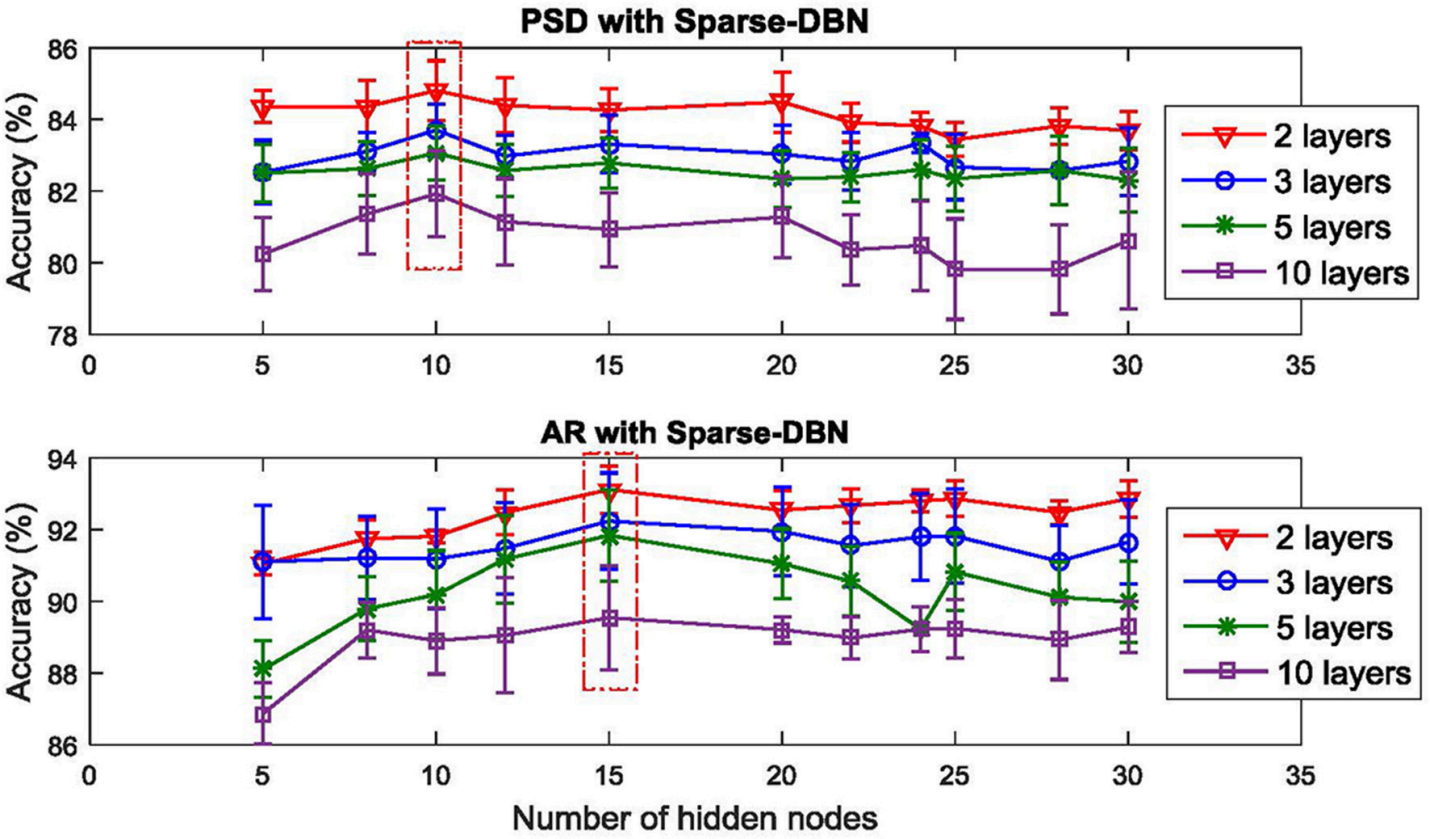
**Plot of the optimal number hidden nodes and layers**.

Using the classification results from the validation set, the optimal number of hidden neurons of the sparse-DBN is shown in Figure 5. For the PSD feature extraction, using 10 hidden nodes resulted in the best classification performance. For the AR feature extraction, using 15 hidden nodes produced the best classification performance. These optimal hidden nodes were then used for the training of the network to classify the test set. Also, the results using a different number of layers (2, 3, 5, and 10 layers) are also provided in Figure 5, with the 2 layers (generative mode for the first layer and discriminative mode for second layer) providing the optimal number of layers in this study. This figure shows that using a higher number of layers (3, 5, and 10 layers) results in a lower accuracy compared to results of using only two layers. Therefore, the two layers sparse-DBN was the chosen architecture providing the higher accuracy. The optimal size of sparse-DBN to classify the PSD features of the EEG-based driver fatigue is [128-10-10-2] and the optimal size of sparse DBN to classify the AR feature is [160-15-15-2]. Table 3 shows the results for the classification of the fatigue state vs. alert state using AR feature extractor and sparse-DBN classifier. For a feature extractor comparison and validity of previous result, the result of the classification using PSD feature extractor method is included. Also for classifier comparison, the classification results using original DBN, BNN and ANN are given.

**Table 3:** **Results classification fatigue state vs. alert state for the test set on different feature extractors and classifiers—early stopping approach**.

**Feature extraction methods**	**Classification results**	**Classification methods**			
		**ANN**	**BNN**	**DBN**	**Sparse-DBN**
PSD	TP	782	808	873	919
	FN	264	238	173	127
	TN	731	791	833	855
	FP	315	255	213	191
	Sensitivity (%)	74.8	77.2	83.5	87.9
	Specificity (%)	69.9	75.6	79.6	81.7
	Accuracy (%)	72.3	76.4	81.5	84.8
AR	TP	845	882	950	982
	FN	201	164	96	64
	TN	814	868	946	965
	FP	232	178	100	81
	Sensitivity (%)	80.8	84.3	90.8	**93.9**
	Specificity (%)	77.8	83.0	90.4	**92.3**
	Accuracy (%)	79.3	83.6	90.6	**93.1**

*Bold values signify improved classification results using proposed method*.

First, for the artificial neural network (ANN) classifier: (i) ANN with PSD, for the fatigue data, of a total with 1,046 units of actual fatigue dataset, 782 units were correctly classified as fatigue states (true positive: TP), resulting in a sensitivity of 74.8%. For the alert group, of a total of 1,046 units of actual alert dataset, 731 units of alert data were correctly classified as alert state (true negative: TN), resulting in a specificity of 69.9%. The combination of ANN and PSD resulted in an accuracy of 72.3%, (ii) ANN with AR, for the fatigue group, of a total of 1,046 units of actual fatigue dataset, 845 units of fatigue data were correctly classified as fatigue states (TP), resulting in a sensitivity of 80.8%. For the alert group, of a total of 1,046 units of actual alert dataset, 814 units of alert data were correctly classified as alert states (TN), resulting in a specificity of 77.8%, while the combination of ANN with AR resulted in an improved accuracy of 79.3% compared to ANN with PSD.

Second, for the Bayesian neural networks (BNN) classifier: (i) BNN with PSD achieved an improvement compared to ANN with PSD, and for the fatigue group, of a total of 1,046 units of actual fatigue dataset, 808 units of fatigue data were correctly classified as fatigue states (TP), resulting in a sensitivity of 77.2%. For the alert state, of a total of 1,046 units of actual alert dataset, 791 units of alert data were correctly classified as alert state (TN), resulting in a specificity of 75.6%. The combination BNN with PSD resulted in an accuracy of 76.4%, (ii) BNN with AR achieved an improvement compared to ANN with AR, and ANN with PSD. BNN with PSD, for the fatigue state, of a total of 1,046 units of actual fatigue data, 882 units were correctly classified as fatigue states (TP), resulting in a sensitivity of 84.3%. For the alert state, of a total of 1,046 units of actual alert data, 868 units of alert data were correctly classified as alert states (TN), resulting in a specificity of 83%. The combination BNN with AR resulted in an accuracy of 83.6%.

Third, when using the deep belief network (DBN) classifier: (i) DBN with PSD achieved a further improvement compared to BNN with PSD, ANN with PSD, and ANN with AR; for the fatigue state, of a total of 1,046 units of actual fatigue data, 873 units of fatigue data were correctly classified as fatigue states (TP), resulting in a sensitivity of 83.5%. For the alert state, of a total of 1,046 units of actual alert data, 833 units of alert data were correctly classified as alert state (TN), resulting in a specificity of 79.6%. The combination DBN with PSD resulted in an accuracy of 81.5%, (ii) DBN with AR achieved further improvement compared to BNN with AR, ANN with AR, DBN with PSD, BNN with PSD, and ANN with PSD, for the fatigue state, of a total of 1,046 units of actual fatigue data, 950 units of fatigue data were correctly classified as fatigue states (TP), resulting in a sensitivity of 90.8%. For the alert state, of a total of 1,046 units of actual alert data, 946 units of alert data were correctly classified as alert states (TN), resulting in a specificity of 90.4%. The combination of DBN with AR resulted in an accuracy of 90.6%.

Fourth, using sparse deep belief networks (sparse-DBN): (i) sparse-DBN with PSD achieved additional improvements compared to DBN with PSD, BNN with PSD, ANN with PSD, BNN with AR, and ANN with AR; for the fatigue state, of a total of 1,046 units of actual fatigue data, 919 units of fatigue data were correctly classified as fatigue states (TP), resulting in a sensitivity of 87.9%. For the alert state, of a total of 1,046 units of actual alert dataset, 855 units of alert data were correctly classified as alert state (TN), resulting in a specificity of 81.7%. The combination sparse-DBN with PSD resulted in an accuracy of 84.8%, (ii) sparse-DBN with AR achieved the most superior result to the other classifier and feature extractor combination with the fatigue state, of a total of 1,046 units of actual fatigue data, 982 units of fatigue data were correctly classified as fatigue states (TP), resulting in a sensitivity of 93.9%. For the alert state, of a total of 1,046 units of actual alert data, 965 units of alert data were correctly classified as alert states (TN), resulting in a specificity of 92.3%. The combination sparse-DBN with AR resulted in best accuracy of 93.1% compared to the other classifier and feature extractor combinations.

## Discussion

In summary, using the PSD feature extractor: (i) compared to the ANN classifier, the sparse-DBN classifier improved the classification performance with sensitivity by 13.1% (from 74.8 to 87.9%), specificity by 11.8% (from 69.9 to 81.7%), and accuracy by 12.5% (from 72.3 to 84.8%); (ii) compared to the BNN classifier, the sparse-DBN resulted in improved performance indicators for sensitivity by 10.7% (from 77.2 to 87.9%), specificity by 6.1% (from 75.6 to 81.7%), and accuracy by 8.4% (from 76.4 to 84.8%); (iii) compared to the DBN classifier, the sparse-DBN resulted in improved performance indicators for sensitivity by 4.4% (from 83.5 to 87.9%), specificity by 2.1% (from 79.6 to 81.7%), and accuracy by 3.3% (from 81.5 to 84.8%).

Further, using the AR feature extractor: (i) compared to the ANN classifier, the sparse-DBN classifier improved the classification performance with sensitivity by 13.1% (from 80.8 to 93.9%), specificity by 14.5% (from 77.8 to 92.3%), and accuracy by 13.8% (from 79.3 to 93.1%); (ii) compared to the BNN classifier, the sparse-DBN resulted in improved performance indicators for sensitivity by 9.6% (from 84.3 to 93.9%), specificity by 9.3% (from 83.0 to 92.3%), and accuracy by 9.5% (from 83.6 to 93.1%); (iii) compared to the DBN classifier, the sparse-DBN resulted in improved performance indicators for sensitivity by 3.1% (from 90.8 to 93.9%), specificity by 1.9% (from 90.4 to 92.3%), and accuracy by 2.5% (from 90.6 to 93.1%).

The result of sensitivity (TPR) and specificity (TNR) analyses can also be viewed as the false positive rate (FPR = 1−specificity) and false negative rate (FNR = 1−sensitivity). The FPR is the rate of the non-fatigue (alert) state being incorrectly classified as fatigue state. The FNR is the rate of fatigue state being incorrectly classified as an alert state. As a result, the proposed classifier (sparse-DBN) with the AR feature extractor resulted in a sensitivity (TPR) of 93.9%, FNR of 6.1%, specificity (TNR) of 92.3%, and FPR of 7.7%. For a real-time implementation, an additional debounce algorithm could be implemented. By adding a debounce component, it masks multiple consecutive false positive detection that may decrease the FPR (Bashashati et al., 2006). The real-time implementation with a debounce algorithm will be a future direction in this area of our study.

For the early stopping classifier comparison, a *k*-fold cross-validation, a popular method for EEG machine learning, is evaluated as well (Billinger et al., 2012). As a result, this study used *k*-fold cross-validation (*k* = 3) with the mean value of 10 results of accuracies on each fold. A total of 6,278 datasets were divided into 3-folds (first-fold = 2,093 sets, second-fold = 2,093 sets, and third-fold = 2,092 sets). Overall, the mean value accuracy of 3-folds was reported. Table 4 shows results using *k*-fold cross validation approach with the chosen AR feature extraction and different classifiers. The result shows that the mean accuracy using the *k*-fold cross validation approach is comparable to the early stopping approach with the proposed classifier of sparse-DBN as the best classifier (94.8% ± 0.011 of sensitivity, 93.3% ± 0.012 of specificity, and 94.1% ± 0.011 of accuracy) and followed by DBN (90.9% ± 0.005 of sensitivity, 90.5% ± 0.005 of specificity, and 90.7% ± 0.005 of accuracy), BNN (84.8% ± 0.012 of sensitivity, 83.6% ± 0.015 of specificity, and 84.2% ± 0.014 of accuracy), and ANN (81.4% ± 0.010 of sensitivity, 78.4% ± 0.012 of specificity, and 79.9% ± 0.011 of accuracy).

**Table 4:** **Results of classification accuracy fatigue state vs. alert state with chosen AR feature extractors and different classifiers—***k***-fold cross validation (3-folds) approach**.

**Classification results**	**Classification methods**			
	**ANN (Mean ± *SD*)**	**BNN (Mean ± *SD*)**	**DBN (Mean ± *SD*)**	**Sparse-DBN (Mean ± *SD*)**
TP	852.0 ± 10.583	888.0 ± 13.229	951.3 ± 4.933	992 ± 11.930
FN	194.7 ± 10.408	158.7 ± 13.051	95.3 ± 4.726	54.3 ± 11.719
TN	820.3 ± 13.051	874.7 ± 15.308	947.0 ± 5.292	976.0 ± 12.288
FP	225.7 ± 13.051	171.3 ± 15.308	99.0 ± 5.292	70.0 ± 12.288
Sensitivity	81.4% ± 0.010	84.8% ± 0.012	90.9% ± 0.005	**94.8%** ± 0.011
Specificity	78.4% ± 0.012	83.6% ± 0.015	90.5% ± 0.005	**93.3%** ± 0.012
Accuracy	79.9% ± 0.011	84.2% ± 0.014	90.7% ± 0.005	**94.1%** ± 0.011

*Bold values signify improved classification results using proposed method*.

One-way ANOVA was used to compare the four classifiers (ANN, BNN, DBN, and sparse-DBN) and the resultant *p*-value was 9.3666e-07. This *p*-value corresponding to the *F*-statistic of one-way ANOVA is much lower than 0.05, suggesting that one or more classifiers are significantly different for which Tukey's HSD test (Tukey−Kramer method) was used to detect where the differences were. The critical value of the Tukey−Kramer HSD *Q* statistic based on the four classifiers and *v* = 8 degree of freedom for the error term, were significance levels of α = 0.01 and 0.05 (*p*-value). The critical value for *Q*, for α of 0.01 (*Q*^α^^= 0.01^) is 6.2044 and the critical value for *Q* for α of 0.05 (*Q*^α^^= 0.05^) is 4.5293. The Tukey HSD Q-statistic (*Q*_*i, j*_) values were calculated for pairwise comparison of the classifiers. In each pair, the statistical significance is found when *Q*_*i, j*_ is more than the critical value of *Q*. Table 5 presents the Tukey HSD Q-statistic (*Q*_*i, j*_) and Tukey HSD *p*-value and Tukey HSD inference of the pairwise comparisons. The results in Table 5 show all six pairwise combinations reached statistical significance (^*^p < 0.05 and ^**^p < 0.01). In addition, to compare the proposed classifier (sparse-DBN) and other classifiers (DBN, BNN, ANN), the sparse-DBN vs. DBN resulted in a *p*-value of 0.021 (^*^p < 0.05), while sparse-DBN vs. BNN and sparse-DBN vs. ANN resulted in a *p*-value of 0.001 (^**^p < 0.01).

**Table 5:** **Result of Statistical significance of Tukey–Kramer HSD in pairwise comparison**.

**Pairwise comparison**	**Tukey HSD *Q*-statistic**	**Tukey HSD *p*-value**	**Tukey HSD inference**
Sparse DBN vs. DBN	5.376	0.021	^*^p < **0.05**
Sparse DBN vs. BNN	15.795	0.001	^**^p < **0.01**
Sparse DBN vs. ANN	22.733	0.001	^**^p < **0.01**
DBN vs. BNN	10.419	0.001	^**^p < 0.01
DBN vs. ANN	17.357	0.001	^**^p < 0.01
BNN vs. ANN	6.938	0.005	^**^p < 0.01

*Bold values signify statistical significance of proposed method vs. other methods*.

* *p < 0.05 statistically significant*.

** *p < 0.01 statistically highly significant*.

Overall, the combination of the AR feature extractor and sparse-DBN achieved the best result with improved sensitivity, specificity and accuracy for the classification fatigue vs. alert states in a simulated driving scenario.

**Figure 6:**
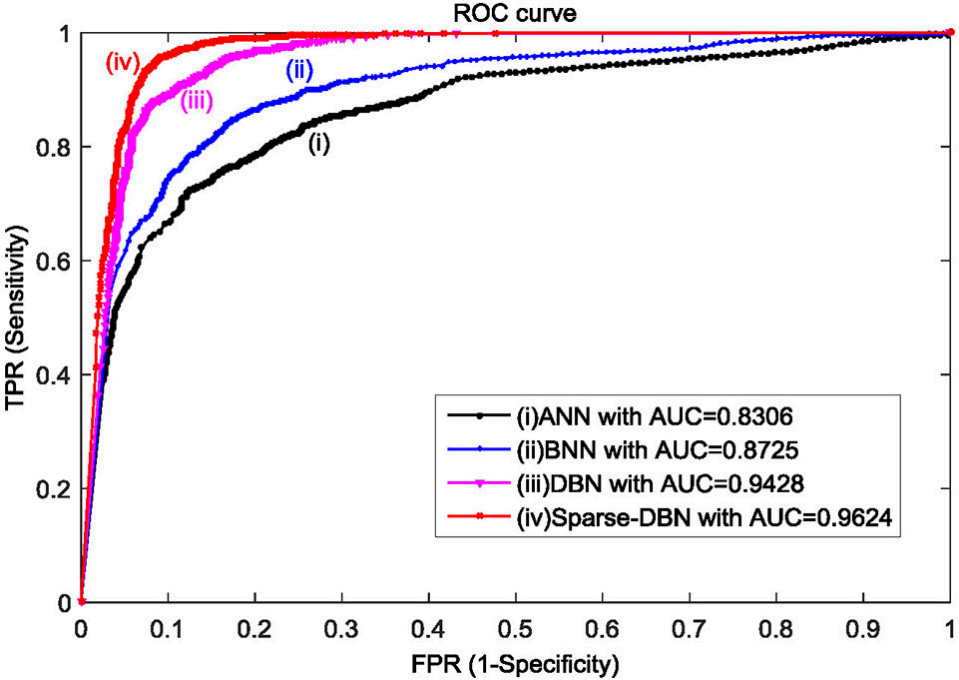
**ROC plot with AUC values for AR feature extractor and ANN, BNN, DBN, and sparse-DBN classifiers of early stopping (hold-out cross-validation) technique**.

Figure 6 shows the results displayed in the receiver operating characteristic (ROC) curve analyses with AR feature extractor and ANN, BNN, DBN, and sparse-DBN classifiers of early stopping (hold-out cross-validation) techniques. The ROC graph is a plot of true positive rate or sensitivity (TPR) on the Y axis and false positive rate (FPR) or 1–specificity on the X-axis by varying different threshold ratios as the sweeping variable. A random performance of a classifier would have a straight line connecting (0, 0) to (1, 1). A ROC curve of the classifier appearing in the lower right triangle suggest it performs worse than random guessing and if the ROC curve appears in the upper left, the classifier is believed to have a superior performance classification (Huang and Ling, 2005; Castanho et al., 2007). All ROC curves in Figure 6 for ANN, BNN, DBN, and sparse-DBN classifier shows the curves plotted in the upper left or above random guess classification. The result also shows that the ROC curve for sparse-DBN classifier achieved the best upper left curve compared to DBN, BNN, and ANN.

The areas under the curve of ROC (AUROC) were also computed to evaluate quantitatively the classification performance. AUROC represents the probability that the classifier will rank a randomly chosen positive example higher than a randomly chosen negative example, and it exhibits several interesting properties compared to accuracy measurement (Huang and Ling, 2005). The AUROC value lies between 0 and 1 with a higher AUROC value indicating a better classification performance. Figure 6 shows that the classifier using sparse-DBN and AR feature extractor achieved the best performance result with the highest AUROC of 0.9624 compared to original DBN classifier with AUROC of 0.9428, BNN classifier with AUROC 0.8725, and conventional ANN with AUROC of 0.8306.

**Figure 7:**
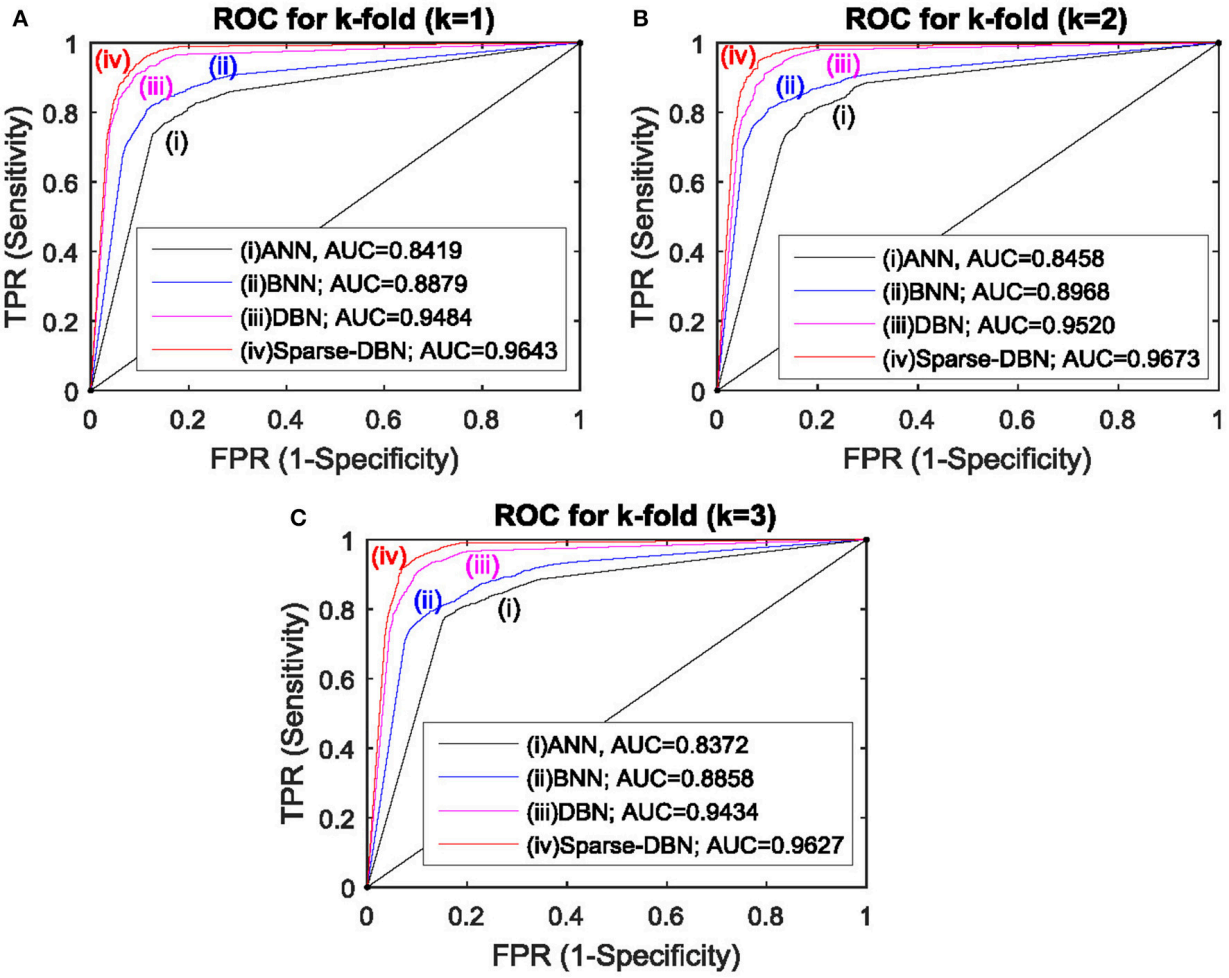
**ROC plot with AUC values for AR feature extractor and ANN, BNN, DBN, and sparse-DBN classifiers of ***k***-fold cross validation (***k*** = 3) technique. (A)** ROC plot with AUC value for 1st fold. **(B)** ROC plot with AUC value for 2nd fold. **(C)** ROC plot with AUC value for 3rd fold.

Figure 7 shows the results displayed in the receiver operating characteristic (ROC) curve analyses with AR feature extractor and ANN, BNN, DBN, and sparse-DBN classifiers of *k*-fold cross-validation (3-folds) technique with three subplots for each fold. Similar with the ROC plot from the hold-out cross validation technique, all ROC curves in Figure 7 for ANN, BNN, DBN, and sparse-DBN classifier shows the curves plotted in the upper left or above random guess classification, and the ROC curve for the sparse-DBN classifier again had best upper left curve compared to DBN, BNN, and ANN. For the area under the curve analysis, in first-fold (*k* = 1), sparse-DBN achieved the best AUROC of 0.9643 compared to DBN classifier with AUROC of 0.9484, BNN classifier with AUROC of 0.8879, and ANN classifier with AUROC of 0.8419. For second-fold (*k* = 2), the sparse-DBN achieved the best AUROC of 0.9673 compared to DBN classifier with AUROC of 0.9520, BNN classifier with AUROC of 0.8968, and ANN classifier with AUROC of 0.8458. For third-fold (*k* = 3), the sparse-DBN achieved the best AUROC of 0.9627 compared to DBN classifier with AUROC of 0.9434, BNN classifier with AUROC of 0.8858, and ANN classifier with AUROC of 0.8372.

Our previous work in Chai et al. (2016) showed a promising result with the inclusion of an additional pre-processing component using a recent independent component analysis (ICA) algorithm, AR feature extractor and BNN classifier. However, it was concluded that the performance of the classification needed to be improved. The findings presented in this paper, strongly suggests that the use of an AR feature extractor provides superior results compared to PSD method, and also extends further the study by improving the reliability including the sensitivity, specificity, and accuracy using sparse-DBN classifier in combination with the AR feature extractor, even without the need to include the ICA pre-processing component.

Using chosen classifier parameters, Table 6 shows the comparison of computation times between the proposed classifier (sparse-DBN) and other classifiers (ANN, BNN, and DBN). The computational time is estimated using the MATLAB *tic*/*toc* function, where the *tic* function was called before the program and the *toc* function afterward on the computer (Intel Core i5−4570 processor 3.20 GHz, 8-GB RAM). The result shows that for the training time, the sparse-DBN required 169.23 ± 0.93 s which was slower compared to other classifiers (86.79 ± 0.24 s for DBN, 55.82 ± 2.77 s for BNN and 24.02 ± 1.04 for ANN). In terms of the testing (classification) time, all classifiers required the same amount of time of 0.03 s or less than a second to complete the task. Although the proposed sparse-DBN required more time to complete the training process, the classifier was able to perform as fast as other classifiers during the testing process. The reason that the testing times of the classifier are comparable to each other was because, after the training process, the final weights were used as constants and in the classification process all classifiers used the same ANN feed-forward classification routine. For the operation of real-time classification, there is no necessity to perform the classifier training again. The classifier just needs to compute the feed forward ANN routine with the saved weight parameters. Thus, sparse-DBN classification time in the runtime mode (execution) is fast, taking less than a second.

**Table 6:** **Comparison of the training time and testing time for different classifiers**.

**Classifiers**	**Training time (s) (Mean ± *SD*)**	**Testing time (s) (Mean ± *SD*)**
ANN	24.02 ± 1.04	0.0371 ± 0.0023
BNN	55.82 ± 2.77	0.0381 ± 0.0082
DBN	86.79 ± 0.24	0.0334 ± 0.0016
Sparse-DBN	169.23 ± 0.93	0.0385 ± 0.0043

The potential future direction of this research includes: (i) real-time driver fatigue with the active transfer learning approach for new user adaptation (Wu et al., 2014; Marathe et al., 2016; Wu, 2016), (ii) improvement of the classification result through an intelligent fusion algorithm, and (iii) testing the efficacy of hybrid driver fatigue detection systems using a combination of physiological measurement strategies known to be related to fatigue status, such as brain signal measurement using electroencephalography (EEG), eye movement and facial tracking systems using camera and electrooculography (EOG), and heart rate variability measurement using electrocardiography (ECG).

## Conclusions

In this paper, the EEG-based classification of fatigue vs. alert states during a simulated driving task was applied with 43 participants. The AR was used for feature extractor and the sparse-DBN was used as a classifier. For comparison, the PSD feature extractor and ANN, BNN, original DBN were included.

Using the early stopping (hold-out cross validation) evaluation, the results showed that for a PSD feature extractor, the sparse-DBN classifier achieved a superior classification result (sensitivity at 87.9%, specificity at 81.7%, and accuracy at 84.8%) compared to the DBN classifier (sensitivity at 83.5%, specificity at 79.6%, and accuracy at 81.6%), BNN classifier (sensitivity at 77.2%, specificity at 75.6%, and accuracy at 76.4%), and ANN classifier (sensitivity at 74.8%, specificity at 69.9%, and accuracy at 72.3%). Further, using an AR feature extractor and the sparse-DBN achieves a significantly superior classification result (sensitivity at 93.9%, specificity at 92.3%, and accuracy at 93.1% with AUROC at 0.96) compared to DBN classifier (sensitivity at 90.8%, specificity at 90.4%, and accuracy at 90.6% with AUROC at 0.94), BNN classifier (sensitivity at 84.3%, specificity at 83%, and accuracy at 83.6% with AUROC at 0.87) and ANN classifier (sensitivity at 80.8%, specificity at 77.8%, and accuracy at 79.3% with AUROC at 0.83).

Overall the findings strongly suggest that a combination of the AR feature extractor and sparse-DBN provides a superior performance of fatigue classification, especially in terms of overall sensitivity, specificity and accuracy for classifying the fatigue vs. alert states. The *k*-fold cross-validation (*k* = 3) also validated that the sparse-DBN with the AR features extractor is the best algorithm compared to the other classifiers (ANN, BNN, and DBN), confirmed by a significance of a *p* < 0.05.

It is hoped these results provide a foundation for the development of real-time sensitive fatigue countermeasure algorithms that can be applied in on-road settings where fatigue is a major contributor to traffic injury and mortality (Craig et al., 2006; Wijesuriya et al., 2007). The challenge for this type of technology to be implemented will involve valid assessment of EEG and fatigue based on classification strategies discussed in this paper, while using an optimal number of EEG channels (i.e., the minimum number that will result in valid EEG signals from relevant cortical sites) that can be easily applied. These remain the challenges for detecting fatigue using brain signal classification.

## Author contributions

RC performed all data analysis and wrote the manuscript. SL, PS, GN, and TN advised the analysis and edited the manuscript. YT and AC conceptualized the experiment and edited the manuscript. HN supervised the study, advised the analysis, and edited the manuscript.

## Funding

This study is funded by “non-invasive prediction of adverse neural events using brain wave activity” from Australian Research Council (DP150102493).

## Ethics statement

Human subjects were given a consent form, which described the experimental procedure and any risks involved (which were minimal). After reading the form, human subjects were asked if they had any questions. Next, human subjects signed the consent form, and then the investigator signed the consent form. The consent forms were stored in a secure filling cabinet in the laboratory.

### Conflict of interest statement

The authors declare that the research was conducted in the absence of any commercial or financial relationships that could be construed as a potential conflict of interest. The reviewer SL and handling Editor declared their shared affiliation, and the handling Editor states that the process nevertheless met the standards of a fair and objective review.
